# 4,6-Dichloro-5-Nitrobenzofuroxan: Different Polymorphisms and DFT Investigation of Its Reactivity with Nucleophiles

**DOI:** 10.3390/ijms222413460

**Published:** 2021-12-15

**Authors:** Elena Chugunova, Nurgali Akylbekov, Alexey Dobrynin, Alexander Burilov, Carla Boga, Gabriele Micheletti, Vincenzo Frenna, Edoardo Jun Mattioli, Matteo Calvaresi, Domenico Spinelli

**Affiliations:** 1 Arbuzov Institute of Organic and Physical Chemistry, FRC Kazan Scientific Center, Russian Academy of Sciences, Akad. Arbuzov st. 8, 420088 Kazan, Russia; aldo@iopc.ru (A.D.); burilov_2004@mail.ru (A.B.); 2 Laboratory of Plant Infectious Diseases, FRC Kazan Scientific Center of Russian Academy of Sciences, Lobachevskogo st. 2/31, 420111 Kazan, Russia; 3 Laboratory of Engineering Profile “Physical and Chemical Methods of Analysis”, Korkyt Ata Kyzylorda University, Aitekebie str. 29A, Kyzylorda 120014, Kazakhstan; nurgali_089@mail.ru; 4 Institute of Radio Electronics, Photonics and Digital Technologies, Kazan National Research Technical University, 10 Karl Marx Str., 420111 Kazan, Russia; 5 Department of Industrial Chemistry ‘Toso Montanari’ ALMA MATER STUDIORUM, Università di Bologna, Viale del Risorgimento 4, 40136 Bologna, Italy; carla.boga@unibo.it; 6 Department STEBICEF, University of Palermo, Ed.17, Viale delle Scienze, 90128 Palermo, Italy; vincenzo.frenna@unipa.it; 7 Department of Chemistry ‘G. Ciamician’ ALMA MATER STUDIORUM, Università di Bologna, Via Selmi 2, 40126 Bologna, Italy; edoardojun.mattioli2@unibo.it (E.J.M.); matteo.calvaresi3@unibo.it (M.C.)

**Keywords:** benzofuroxans, X-ray structures, polymorphism, nucleophilic substitutions, DFT calculations

## Abstract

This research focuses on the X-ray structure of 4,6-dichloro-5-nitrobenzofuroxan **1** and of some of its amino derivatives (**4a, 4e, 4g,** and **4l**) and on DFT calculations concerning the nucleophilic reactivity of **1**. We have found that by changing the solvent used for crystallization, it is possible to obtain 4,6-dichloro-5-nitrobenzofuroxan (**1**) in different polymorphic structures. Moreover, the different torsional angles observed for the nitro group in **1** and in its amino derivatives (**4a**, **4e**, **4g,** and **4l**) are strictly dependent on the steric hindrance of the substituent at C-4. DFT calculations on the course of the nucleophilic substitution confirm the role of the condensed furoxan ring in altering the aromaticity of the carbocyclic frame, while chlorine atoms strongly influence the dihedral angle and the rotational barrier of the nitro group. These results corroborate previous observations based on experimental kinetic data and give a deep picture of the reaction with amines, which proceeds via a “non-aromatic” nucleophilic substitution.

## 1. Introduction

In line with our interest in the study of the reactivity/activity of heterocyclic compounds, we thoroughly investigated the chemical/biological behavior of several classes of heterocycles containing nitrogen, oxygen, and sulphur atoms. Thus, we examined ring-into-ring rearrangements [1,2,3,4], reactivity with nucleophiles [5,6,7], ring-opening [8,9], and so on [10,11,12]. Moreover, considering that in the last few decades, the interest of chemists has been deeply focused on the pharmaceutical/pharmacological activities of several heterocyclic compounds [13,14,15], we evaluated some of their cardiovascular [16], antitumor [17], or anti-MDR1 [18] effects.

The study of biologically active compounds is one of the most interesting and rapidly developing branches of organic chemistry. The range of synthetic chemical compounds used in medicine and agriculture is constantly expanding, and this requires a continuous search for new substances with potential biological activity, and, consequently, the development of convenient, fresh methods for their synthesis [19,20,21,22].

Benzofuroxans (benzo[*c*][1,2,5]oxadiazole 1-oxides) occupy a relevant place in the variety of synthetic biological active compounds and are also of interest from a mechanistic point of view [23,24,25,26,27]. Benzofuroxans exhibit a wide spectrum of biological activities: they behave as UV-protective [28], antituberculosis [29], antileukemic, immunosuppressive [30], anti-infective [31], antibacterial [32], antifungal [33], insecticidal [34], and antiparasitic (antiplasmodial and trypanocidal) agents [35], calcium channel modulators [36], and monoamine oxidase inhibitors [37]. Benzofuroxans also find applications in different fields, such as pharmaceuticals, veterinary medicine, agricultural chemistry, and dyes [28,38,39,40,41]. 

Moreover, since the discovery of the physiological role of nitric oxide as a second messenger, the issue of identifying new ways to stimulate its formation in the body has become a relevant target [42,43,44,45]. The presence of the *N*-oxide fragment in the molecule of benzofuroxans has undoubtedly caused interest in the study of this class of compounds as a probable nitric oxide donor agent [46,47,48].

Derivatives of benzofuroxan can also be used as high-energy materials [49]. The benzofuroxan scaffold is moreover a promising starting point for the synthesis of different heteroaromatic and polycyclic heterocyclic compounds [50]. 

**Figure 1:**
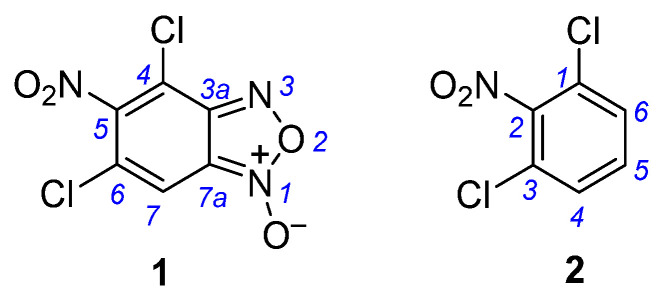
Structure of 4,6-dichloro-5-nitrobenzofuroxan (**1**) and of 1,3-dichloro-2-nitrobenzene (**2**).

Accordingly, some of us are conducting a long-term study of new syntheses of benzofuroxan derivatives, their chemical reactivity, biological activity, and the possibility of their use in the biological/pharmacological fields [7,23,27,28,51]. Among the benzofuroxans used in our previous works, 4,6-dichloro-5-nitro-benzofuroxan (**1**) (Figure 1) is one of the most interesting chemical platforms because it easily reacts with various nucleophiles, leading to the formation of products with very interesting antimicrobial activity. Over the years, some researchers have synthesized, described, and studied the biological activity of more than 80 compounds deriving from it [52,53]. However, as some recent results have shown, this compound is of interest not only for its ability to give biologically active substances, but also for its chemistry, showing interesting reactivity with some nucleophiles [7,23,53] and also giving polymorphic forms of crystals during crystallization.

**Scheme 1:**
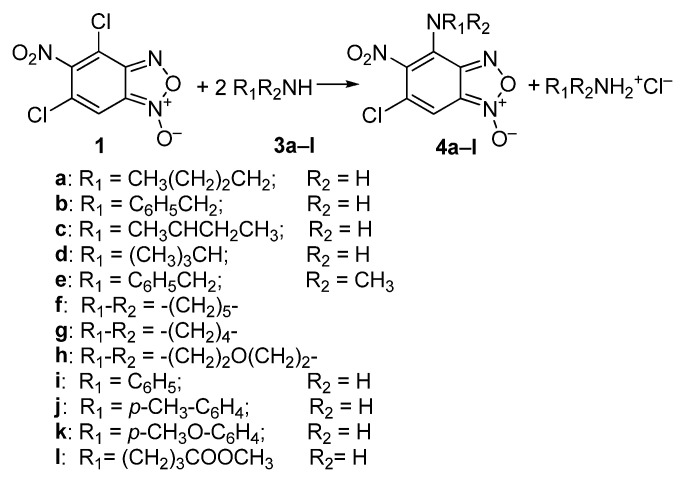
Nucleophilic reactivity of **1** with amines.

We recently investigated the nucleophilic reactivity of 4,6-dichloro-5-nitrobenzofuroxan (**1**) carrying out a kinetic study of its behavior in methanol and in toluene with a “fan” of amines (Scheme 1: **3a**–**k**; aliphatic, primary and secondary, with low or high steric requirements; aromatic) [7], thus covering a wide range of basicity/nucleophilicity and steric requirements [54,55,56,57,58] and also gaining interesting information on the course of the reaction.

Interestingly, the compound **1**, which can be considered as deriving from 1,3-dichloro-2-nitrobenzene (**2**) by condensation of a furoxan ring at a C_4_–C_5_ bond, shows chemical behavior that is quite different from that exhibited by **2** [59,60]. If we compare the structures of **1** and **2,** we can observe some resemblances, but also some significant differences.

In both, the presence of two chlorine atoms in the 1,3-relationship constrains the nitro group at C_2_ out of the carbocyclic plane, greatly lowering its electron-withdrawing power. That means a kind of secondary steric effect will also occur [61], thus lowering the ability of the nitro group and activating a nucleophilic substitution process. This event causes a very low reactivity of **2** with nucleophiles.

Moreover, we can observe that in **2,** the carbocyclic ring has an aromatic character and that the two chlorine atoms are chemically equivalent (this means that one of them can be indifferently substituted by the action of a nucleophile), while in **1,** the carbocyclic ring (because of the condensation with the furoxan ring at the C_4_–C_5_ bond of **2**) does not have an aromatic character and the two chlorine atoms are not chemically equivalent; accordingly, we have observed that only the chlorine at C_4_ can be substituted by nitrogen nucleophiles [7]. Finally, the condensed furoxan ring strongly affects the global electrophilic character of **1** and then its ability to react with nucleophiles. 

**Figure 2:**
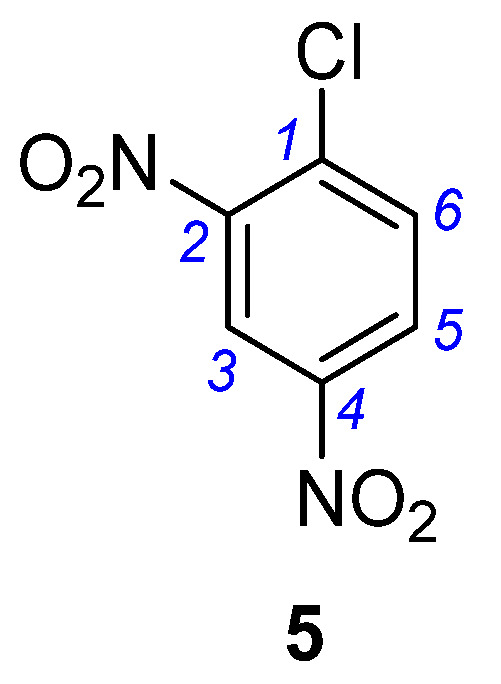
2,4-Dinitrochlorobenzene (**5**).

As a matter of fact, while **2** shows a very low reactivity with nitrogen nucleophiles (aliphatic, as well as aromatic amines) [59,60], **1**, in contrast, shows high reactivity [7] with the same amines, comparable to that of 2,4-dinitrochlorobenzene (**5**, Figure 2) [62].

This peculiar behavior of 4,6-dichloro-5-nitrobenzofuroxan (**1**) can be explained by taking into account two factors: (1) the strong electron-attracting effect of the condensed furoxan ring (which behaves as a strong electron-attracting group); and (2) the low aromatic character of the benzofuroxan ring, as indicated by its relatively low value of the Bird aromaticity index (81.0, perhaps the lowest I_A_ reported for a benzocondensed aromatic system) [63,64,65].

**Figure 3:**
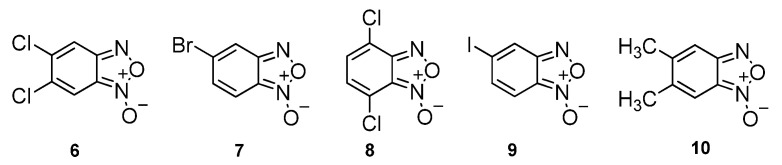
Representative benzofuroxans for which the phenomenon of crystal polymorphism was observed.

To confirm the above considerations and gain further information on the structural characteristics of 4,6-dichloro-5-nitrobenzofuroxan (**1**) and of some of its amino derivatives, we have now collected new data concerning the X-ray structure of **1** and of some of its products of substitution with amines (**4a**, **4e**, **4g**, and **4l**). The literature reports examples of polymorphism for some benzofuroxan derivatives (Figure 3).

For example, three different polymorphs (A, B, and C) were observed for 5,6-dichlorobenzofuroxan (**6**) [66]. All had disorders about a two-fold or pseudo-two-fold axis and were packed in ribbons in a head-to-tail fashion, such that the chlorine atoms of one molecule are close to the oxygen atoms of the next molecule.

Then, in 2001, it was firstly shown that at low temperatures, 5-bromobenzofuroxan (**7**) transformed into a second polymorph with a unit cell that was twice as large, but with essentially the same packing [67].

Moreover, 4,7-dichlorobenzofuroxan (**8**) and 5-iodobenzofuroxan (**9**) occur in two polymorphic forms [68,69].

At last, it was found that 5,6-dimethylbenzofuroxan (**10**) exists in four polymorphic forms that are polytypes of each other [70]. Each polymorph of 5,6-dimethylbenzofuroxan contains molecules disordered about pseudo-two-fold axes and arranged head-to-tail in ribbons, with the ribbons forming approximate planar layers held together by weak C—H···N and C—H···O interactions.

In addition, by using DFT computations, we collected data concerning the rotational barrier of the investigated compounds in solution, their electronic distribution, and the nucleophilic reactivity of **1**.

## 2. Results

**Figure 4:**
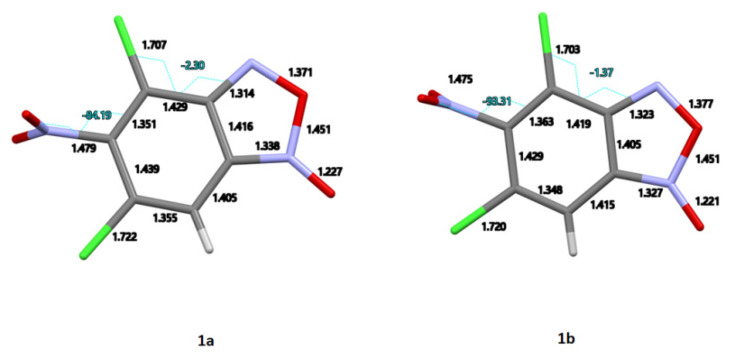
Geometry of **1a** (**left**) and **1b** (**right**).

Looking at 4,6-dichloro-5-nitrobenzofuroxan (**1**) its structure was confirmed by X-rays analysis, evidencing that it exists in two different polymorphs (**1a** and **1b**; see Figure 4) depending on the solvent used for the crystallization. **1a** (Figure 4, left) crystallizes from chloroform/hexane in the triclinic space group *P-1* with one molecule of **1a** per asymmetric unit. In contrast, polymorph **1b** (Figure 4, right) obtained by crystallization from acetone/pentane is an orthorhombic one. It crystallizes in space group *P2_1_2_1_2_1_* with one molecule of **1b** per asymmetric unit.

**Figure 5:**
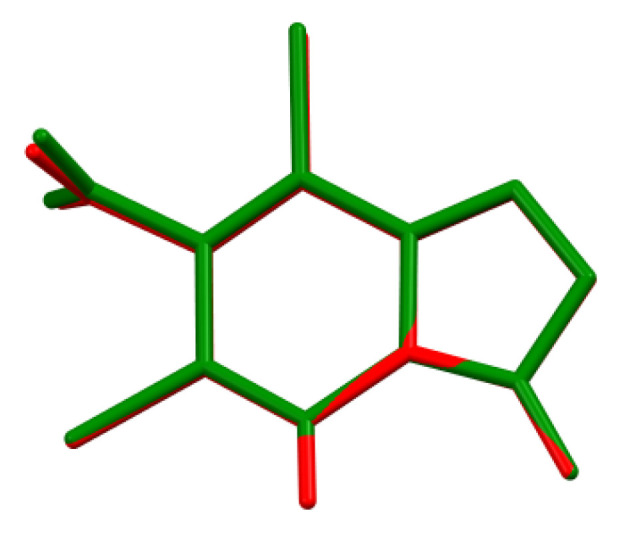
Overlay structures of **1a** (green) and **1b** (red).

The substituted benzofuroxan fragment is planar in both structures, and nitro groups largely deviate from the molecule’s plane on slightly different angles, between −84° and −93° (Figure 5). Interestingly, this datum well confirms our hypothesis [7] that because of the presence of the two adjacent chlorine atoms, the nitro group of **1** “suffers” a kind of secondary steric effect [61], and then it is not able to activate the process of nucleophilic substitution to the best of its ability. Moreover, looking at the C–C bond lengths in the carbocyclic ring of **1**, we received confirmation on our hypothesis [7] that this ring does not have a “real” aromatic character. As a matter of fact, their lengths range from 1.351 Å (for the C_4_–C_5_ bond) to 1.439 Å (for the C_5_–C_6_ bond) in **1a**, and from 1.363 Å (for the C_4_–C_5_ bond) to 1.429 Å (for the C_5_–C_6_ bond) in **1b**. Interestingly, the length variations in the two polymorphs show similar trends (see Figure 5).

**Figure 6:**
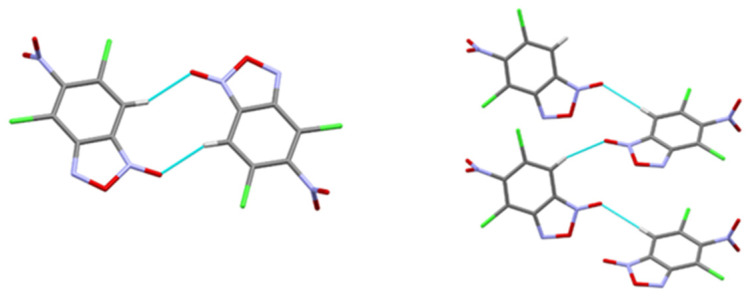
Packing of **1a** (**left**) and **1b** (**right**).

Dimer is formed in **1a** due to intermolecular hydrogen bonds of C–H···O-type between the hydrogen at C_7_ and the oxygen at N_1_ (Figure 6), while an infinite chain along the crystallographic 0α axis is formed in the **1b** (Figure 6).

**Figure 7:**
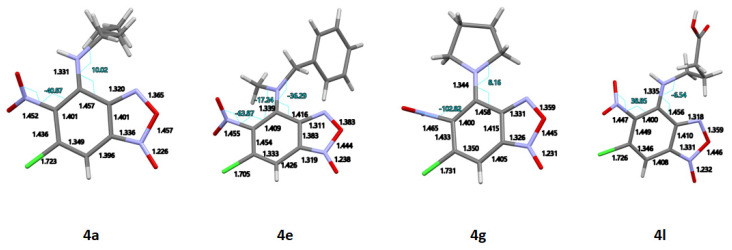
Geometry of **4a**, **4e**, **4g**, and **4l**.

Concerning compounds **4a**, **4e**, **4g [7]**, and **4l** [23], the relevant results of X-ray structure determinations are summarized in Figure 7.

The examination of the structures obtained for **4a**, **4e**, **4g**, and **4l**, compared with that of **1**, requires some comments. Of course, we can anticipate that the entity of the rotation of the nitro group in **1** and in the relevant amines shall depend on the different steric hindrances exerted by the substituents present at C_4_. In fact, we have observed that in all of them, the rotation of the nitro group with respect to the carbocyclic ring is significantly lower than in **1,** and this datum is well in line with provisions. In fact, looking at the substituents at C_4_, ongoing from a chlorine atom (in **1**) to a secondary (in **4e** and in **4g**) and then to a primary amino group (in **4a** and in **4l**), a significant decrease of the steric hindrance occurs, and then a lowering of the out-of-plane rotation of the nitro group must be (and has been) observed. This result depends on the lower steric effect exerted by the three classes of substituents, which causes a lowering of the rotation of the nitro group from −84° or −93° (in **1a** and **1b**) to −64° and −103° (in **4e** and **4g**) and to −41° and 39° (in **4a** and **4l**).

**Figure 8:**
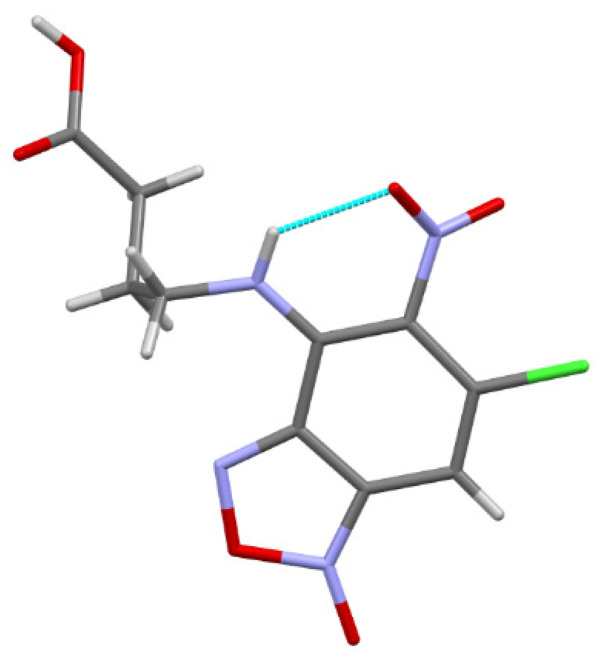
Intramolecular H-bond in **4l**.

**Figure 9:**
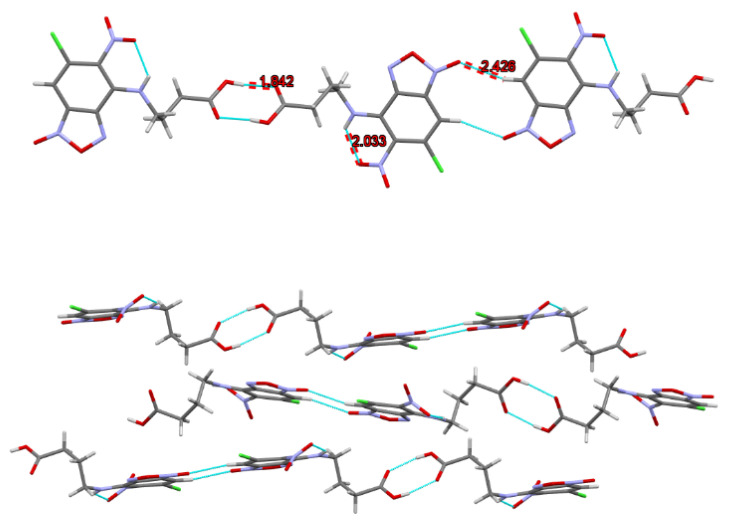
A packing of molecules in crystal of compound **4l**. Only classical hydrogen bonds are observed.

**Figure 10:**
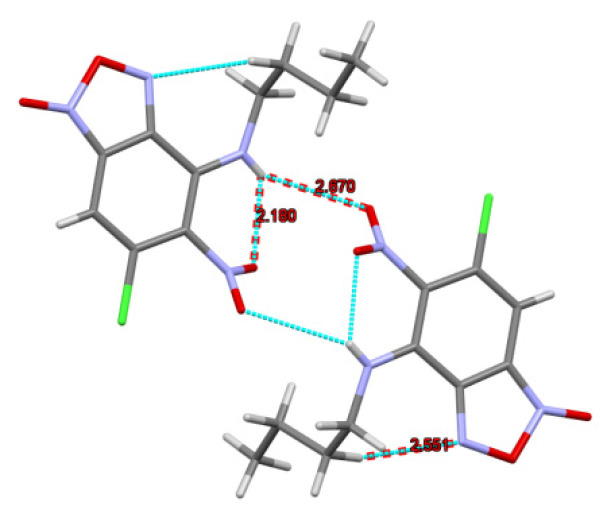
A packing of molecules in crystal of compound **4a**.

**Figure 11:**
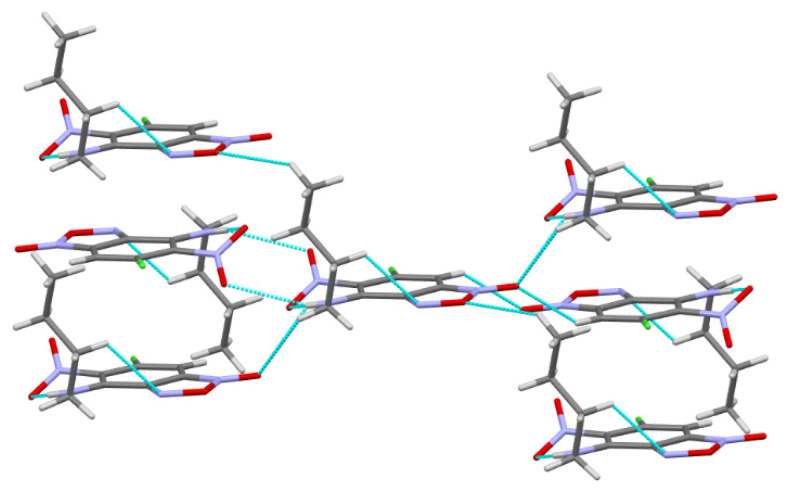
A packing of molecules in crystal of compound **4a**.

The presence of a hydrogen atom linked to the amino nitrogen one in **4a** and in **4l** makes the formation of hydrogen bonds possible with the oxygen atoms of the adjacent nitro group. We have observed different behaviors in the two compounds—in **4l,** we only observed the occurrence of an intramolecular hydrogen bond (Figure 8 and Figure 9), while we noted both an intramolecular and an intermolecular hydrogen bond in **4a** (Figure 10 and Figure 11). This different behavior could be associated to the appearance of electrostatic repulsion between the nitro group and the acceptor carboxyl group, which prevents the approach of two molecules and the occurrence of a multicenter hydrogen bond.

**Figure 12:**
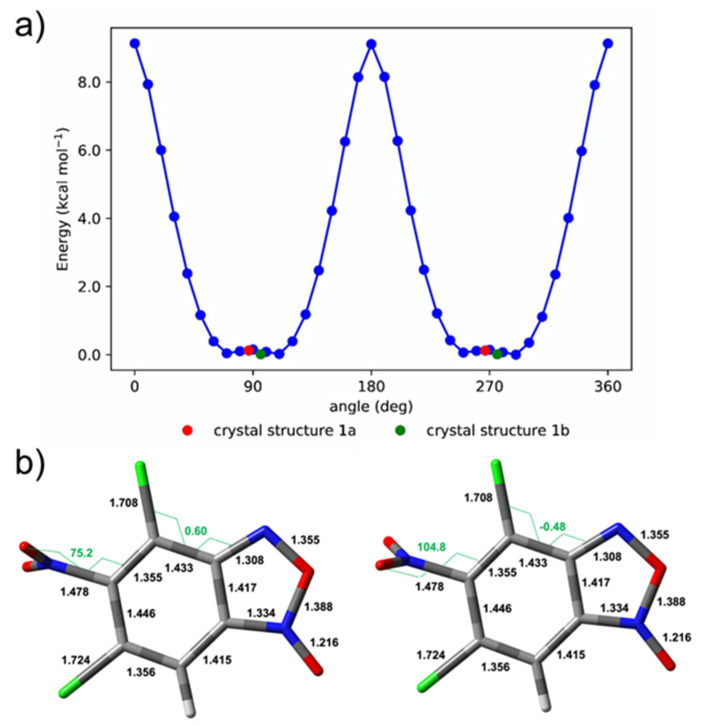
(**a**) Rotational barrier of the nitro group in the 4,6-dichloro-5-nitrobenzofuroxan molecule (**1**) in solution. (**b**) Optimized structures of (**1**) in solution.

To investigate the structural characteristics of the 4,6-dichloro-5-nitrobenzofuroxan molecule in solution, we carried out DFT calculations. Starting from the coordinates of the two polymorphs **1a** and **1b**, and optimizing their structure in solution, they converge toward four degenerate minima (Figure 12). The torsional angles of the nitro group in the minima are 75.2°, 104.8°, 255.2°, and 284.8°. In these geometries, the nitro group is very close to an orthogonal arrangement with respect to the aromatic plane of the molecule. Additionally, in solution, the nitro group of **1** “suffers” a kind of secondary steric effect, because of the presence of the two adjacent chlorine atoms.

The energy necessary to pass from the minima to the conformations assumed by the molecules in the polymorph **1a** and **1b** is minimal (0.15 kcal mol^−1^ for **1a** and 0.04 kcal mol^−1^) due to the flatness of this region of the potential energy surface. The calculated rotational barrier of the nitro group in (**1**) is 9.15 kcal mol^−1^.

To shed light on the role of chlorine substituents in exerting secondary steric effect toward the nitro group, we also calculated the potential energy profiles, related to the torsional angle of the nitro group, on the monochlorinated compounds 6-chloro-5-nitrobenzofuroxan (**11**) and 4-chloro-5-nitrobenzofuroxan (**12**) and on 1,3-dichloro-2-nitrobenzene (**2**) (Figure S1).

Interestingly, in **2,** the dihedral angles of the nitro group in the minima are 74.4°, 105.6°, 254.4°, and 285.6°. These torsional angles are very close to the value observed in **1**. Additionally, the value of the rotational barrier of the nitro group (7.8 kcal mol^−1^) in **2** is in line with the value obtained for **1**. On the contrary, the equilibrium value of the torsional angles of the nitro group, in absence of the second chlorine atom, shifts toward planarity for **11** (52.6°, 129.4°, 232.6°; 309.4°), and even more for **12** (43.5°, 136.1°, 223.5°, 316.1°). More importantly, the absence of the second chlorine atom greatly lowers the rotational barrier of the nitro group (2.6 and 1.3 kcal mol^−1^ for **11** and **12**, respectively). This result confirms the role of the “couple” of chlorine atoms to hinder the rotation of the nitro group.

To investigate how the “condensed” furoxan ring affects the aromaticity of the benzene ring, we analyzed the bond lengths of the optimized structures in (**1**), (**2**), **(11)**, and (**12**).

**Figure 13:**
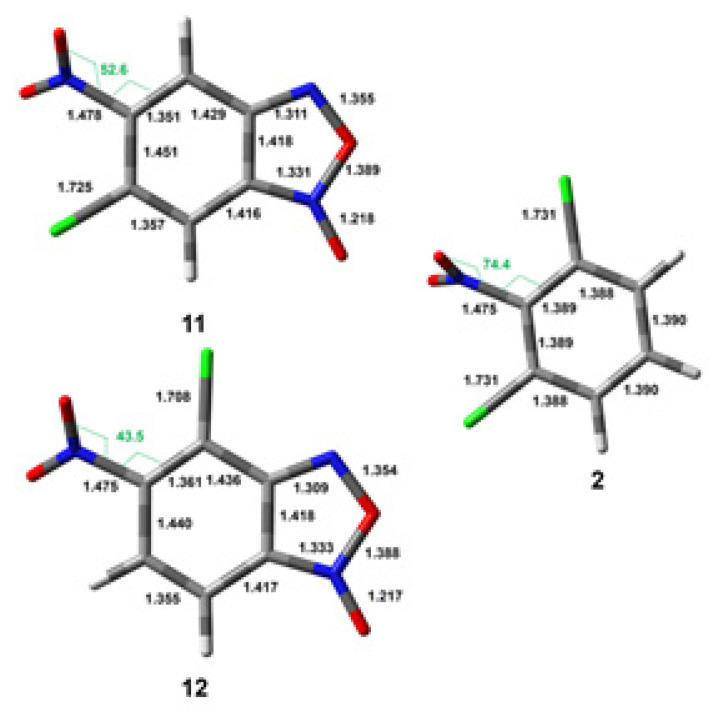
Optimized geometry of **11, 12,** and **2** in solution.

The lack of a second chlorine atom does not affect the low aromatic character of (**1**). In fact, in (**11)** and (**12**), the bond lengths that characterize the carbocyclic moiety do not vary significantly upon removal of the second chlorine atom (Figure 13). We can conclude that the number of chlorine atoms in the ring does not influence its aromaticity.

On the contrary, when the condensed furoxan ring is lacking, as in (**2**) (Figure 13), a clear aromatic character of the benzene moiety also appears when the two chlorines are present as substituents.

In conclusion, DFT calculations confirm the role of the condensed furoxan ring in altering the aromaticity of the carbocyclic frame, while chlorine atoms strongly influence the dihedral angle and the rotational barrier of the nitro group. These results corroborate previous observations based on experimental kinetic data [7].

Moving from structure to reactivity, when we investigated the nucleophilic substitution of 4,6-dichloro-5-nitrobenzofuroxan [7], we observed that in **1,** only the selective substitution of chlorine at C_4_ by nitrogen nucleophiles occurred.

**Figure 14:**
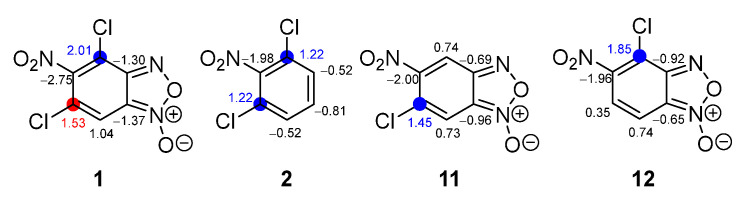
Mulliken partial charges of compounds **1**, **2**, **11**, and **12**. Indicated in blue are the most positive electrophilic carbon atoms; in red are the second most positive electrophilic carbon atoms.

The calculated Mulliken charges (Figure 14) well explain the regioselectivity of the nucleophilic substitution reaction between **1** and the nucleophilic amines. The reactive carbon atom C_4_ is characterized by the largest positive charge (2.01 au). The charge at C_6_ is smaller (1.53 au). The strong electron-withdrawing effect, exerted by the condensed furoxan ring, is clear by comparing the values of the charges in **1** with those of **11** and **12**, and that of **1** with that of **2**.

In addition, our previous work [7] showed that **1** is much more reactive with nitrogen nucleophiles than **2** and had a reactivity similar to that of 2,4-dinitroclorobenzene (**5**) [62].

The Frontier Molecular Orbital (FMO) theory provides support for this evidence. The calculated HOMO-LUMO gap between the HOMO of the nucleophile (pyrrolidine) and the LUMO of the electrophilic compounds **1**, **2,** and **5** is 5.48, 6.61, and 5.89 eV. These gaps agree with experimental reactivity observations where compounds **1** and **5** show similar reactivity (small HOMO-LUMO gap), while compound **2** is characterized by much lower reactivity (large HOMO-LUMO gap).

**Figure 15:**
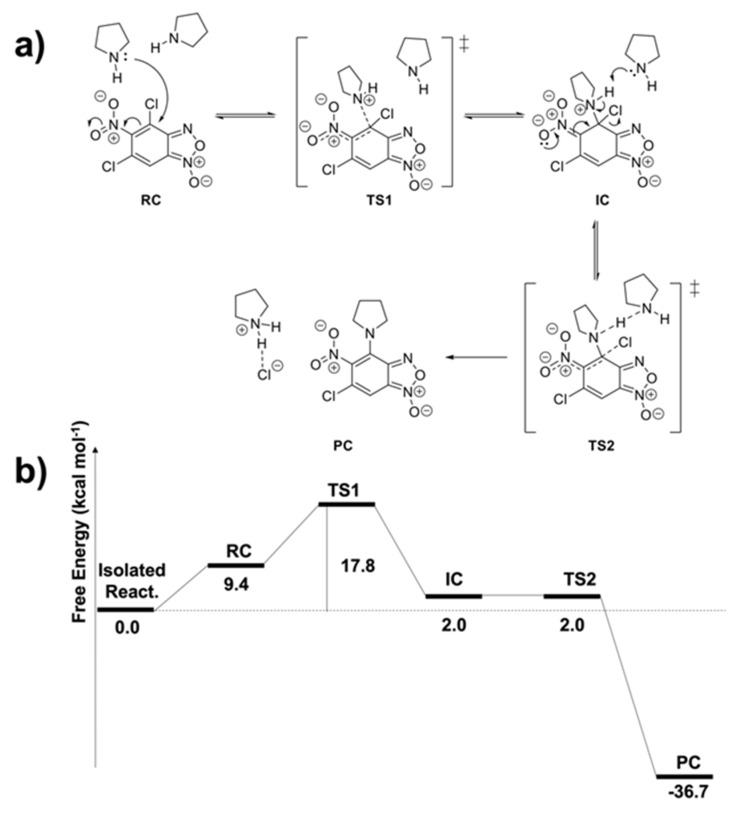
(**a**) Reaction mechanism and (**b**) energy profile (energies in kcal mol^−1^) of the reactions of 4,6-dichloro-5-nitrobenzofuroxan (**1**) with pyrrolidine.

To gain a quantitative description of the reaction mechanism, we calculated the energy profile of the selective nucleophilic substitution of **1** with pyrrolidine. Figure 15 depicts the reaction mechanism, which is a two-step process involving a nucleophilic attack of a pyrrolidine molecule on the reactive C_4_ centre of **1** at the first reversible stage, leading to the formation of the intermediate IC, and then at the second stage, the elimination of the chlorine anion with a concerted proton transfer to a second pyrrolidine molecule leading to the formation of the product PC and pyrrolidinium chloride.

The first step is characterized by an energy barrier of 17.8 kcal mol^−1^, in excellent agreement with experimental kinetic data where the measured activation free energy is 17.7 kcal mol^−1^. The formation of the intermediate IC is a slightly endoergonic process (+2.0 kcal mol^−1^), but the barrierless elimination of the chlorine atom with the concerted proton transfer rapidly leads to the stable product PC (**4g**) that is strongly exoergonic (−36.7 kcal mol^−1^), making the reactive process irreversible in agreement with experimental data.

The addition/elimination process of the pyrrolidine molecule to the 4,6-dichloro-5-nitrobenzofuroxan (**1**) resembles an aza-Michael addition to nitro alkenes, where the nitro group exerts the typical push/pull effect [71]. In fact, during the addition process, an incipient electron flow from the pyrrolidine nitrogen (N_PYR1_) is directed toward the C_4_–C_l_ bond (see RC in Figure 15a), also involving the conjugated nitro group, as clearly observed from data in Table 1, particularly looking at the C_4_–C_5_ and C_5_–N_NO2_ bond lengths. Two-dimensional and three-dimensional representations of the various critical points are given in the SI.

This observation explains how the nitro group supports the addition step of the nucleophilic substitution (pulling effect). This process is additionally enhanced by the fused furoxan ring. In fact, the furoxan ring, as described previously, breaks the aromaticity in the carbocyclic ring, and the C_4_–C_5_ double bond becomes similar to an electron-poor olefin bond. The other bond lengths that characterize the compound **1** are substantially unchanged during the reaction, evidencing the localization of the reactive process.

In TS_2_ the reaction coordinate is dominated by the proton transfer from IC to a pyrrolidine molecule: N_PYR13_–H (1.19 Å) and H–N_PYR21_ (1.44 Å). The proton transfer involves a second pyrrolidine molecule and triggers the barrierless exit of the chloride anion. Again, this reactive process is facilitated by the nitro group (pushing effect) that makes the leaving of the chloride anion a highly favoured process (see IC in Figure 15a). The elimination process restores the geometries of the C_4_–C_5_ and C_5_–N_NO2_ bonds similarly to the reactant, as expected in a substitution process.

## 3. Materials and Methods

### 3.1. General

The following compounds were prepared according with the literature procedures indicated: 4,6-dichloro-5-nitrobenzo[*c*][1,2,5]oxadiazole 1-oxide (**1**) [53], and 4-aminobenzo[*c*][1,2,5]oxadiazole 1-oxides **4a**, **4e**, **4g** [7], and **4l** [23]. 

### 3.2. Crystallographic Analyses

The X-ray diffraction experiments for compounds **1a**, **1b**, **4a**, **4e**, **4g**, and **4l** were carried out on a Bruker KAPPA APEX II CCD diffractometer (graphite-monochromated Mo Kα (0.71073 Å) radiation). Data collection: images were indexed, integrated, and scaled using the APEX2 [72] data reduction package and corrected for absorption using SADABS [73]. The structures of compounds were solved by the direct methods and refined by the anisotropic (isotropic for all H atoms) full-matrix least-squares method against *F*^2^ of all reflections using SHELX [74]. The positions of the hydrogen were calculated geometrically and refined in the riding model.

#### 3.2.1. Crystallographic Data for 4,6-dichloro-5-nitrobenzo[*c*][1,2,5]oxadiazole 1-oxide (**1a**)

C_6_HCl_2_N_3_O_4_, M 250.00, triclinic, *P-1*, *a* 7.124(5), *b* 7.359(5), *c* 8.614(6) Å, *α* 91.478(8), *β* 95.886(8), *γ* 105.257(8)°, *V* 432.7(5) Å^3^, *Z* 2, *D_calcd_* 1.919g·cm^−3^, *μ*(Mo-*Kα*) 0.746 mm^−1^, F(000) 248, (θ 2.4–28.0°, completeness 99.9%), T 150(2) K, orange prism, (0.05 × 0.1 × 0.1) mm^3^, 5691 measured reflections in index range −9 ≤ h ≤ 9, −9 ≤ k ≤ 9, −11 ≤ l ≤ 11, 2082 independent (*R_int_* 0.036), 136 parameters, *R_1_* = 0.0320 (for 1627 observed *I* > 2σ(*I*)), *wR_2_* = 0.0823 (all data), GOOF 1.00, largest diff. peak and hole 0.40 and −0.28 e.A^−3^. 

#### 3.2.2. Crystallographic Data for 4,6-dichloro-5-nitrobenzo[*c*][1,2,5]oxadiazole 1-oxide (**1b**)

C_6_HCl_2_N_3_O_4_, M 250.00, orthorhombic, *P2_1_2_1_2_1_, a* 6.3259(18), *b* 6.4507(18), *c* 21.315(6) Å, *V* 869.8(4) Å^3^, *Z* 4, *D_calcd_* 1.909 g·cm^−3^, *μ*(Mo-*Kα*) 0.742 mm^−1^, F(000) 496, (θ 1.9–27.0°, completeness 100.0%), T 150(2) K, colorless prism, (0.07 × 0.09 × 0.28) mm^3^, 5420 measured reflections in index range −8 ≤ h ≤ 8, −8 ≤ k ≤ 6, −27 ≤ l ≤ 27, 1893 independent (*R_int_* 0.036), 136 parameters, *R_1_* = 0.0326 (for 1684 observed *I* > 2σ(*I*)), *wR_2_* = 0.0652 (all data), GOOF 1.06, largest diff. peak and hole 0.25 and −0.25 e.A^−3^.

#### 3.2.3. Crystallographic Data for 4-(butylamino)-6-chloro-5-nitrobenzo[*c*][1,2,5]oxadiazole 1-oxide (**4a**)

C_10_H_11_ClN_4_O_4_, M 286.68, triclinic, *P-1*, *a* 7.4066(8), *b* 8.4714(11), *c* 10.2782(13) Å, *α* 94.214(6), *β* 93.778(5), *γ* 111.867(5)°, *V* 593.84(13) Å^3^, *Z* 2, *D_calcd_* 1.603g·cm^−3^, *μ*(Mo-*Kα*) 0.340 mm^−1^, F(000) 296, (θ 3.1–26.0°, completeness 99.3%), T 296(2) K, orange prism, (0.17 × 0.28 × 0.61) mm^3^, transmission 0.6687–0.7454, 4954 measured reflections in the index range −6 ≤ h ≤ 9, −10 ≤ k ≤ 10, −12 ≤ l ≤ 12, 2316 independent (*R_int_* 0.038), 173 parameters, *R_1_* = 0.0421 (for 1416 observed *I* > 2σ(*I*)), *wR_2_* = 0.0893 (all data), GOOF 0.92, largest diff. peak and hole 0.23 and −0.26 e.A^−3^. 

#### 3.2.4. Crystallographic Data for 4-(benzyl(methyl)amino)-6-chloro-5-nitrobenzo[*c*][1,2,5]oxadiazole 1-oxide (**4e**)

C_14_H_11_ClN_4_O_4_, M 334.72, monoclinic, *P2_1_/c*, *a* 11.53(2), *b* 10.599(9), *c* 12.047(15) Å, *β* 90.68(12)°, *V* 1472(3) Å^3^, *Z* 4, *D_calcd_* 1.510 g·cm^−3^, *μ*(Mo-*Kα*) 0.286 mm^−1^, F(000) 688, (θ 2.6-30.0°, completeness 76.4%), T 296(2) K, orange prism, (0.12 × 0.20 × 0.31) mm^3^, transmission 0.3945–0.7455, 6389 measured reflections in the index range −16 ≤ h ≤ 15, −13 ≤ k ≤ 15, −13 ≤ l ≤ 15, 3378 independent (*R_int_* 0.340), 210 parameters, *R_1_* = 0.1330 (for 541 observed *I*> 2σ(i)), *wR_2_* = 0.1479 (all data), GOOF 0.89, largest diff. peak and hole 0.19 and −0.20 e.A^−3^. 

#### 3.2.5. Crystallographic Data for 6-chloro-5-nitro-4-(pyrrolidin-1-yl)benzo[*c*][1,2,5]oxadiazole 1-oxide (**4g**)

C_10_H_9_ClN_4_O_4_, M 284.66, triclinic, *P-1*, *a* 8.8447(11), *b* 10.8450(15), *c* 13.5532(18) Å, *α* 93.052(3), *β* 104.591(3), *γ* 108.750(3)°, *V* 1178.5(3) Å^3^, *Z* 4 (2 independent molecules), *D_calcd_* 1.604g·cm^−3^, *μ*(Mo-*Kα*) 0.342 mm^−1^, F(000) 584, (θ 2.5–27.0°, completeness 98.7%), T 296(2) K, orange prism, (0.18 × 0.31 × 0.61) mm^3^, transmission 0.6865–0.7463, 10088 measured reflections in index range −11 ≤ h ≤ 7, −13 ≤ k ≤ 13, −17 ≤ l ≤ 17, 5056 independent (*R_int_* 0.030), 343 parameters, *R_1_* = 0.0486 (for 3439 observed *I* > 2σ(*I*)), *wR_2_* = 0.1546 (all data), GOOF 1.02, largest diff. peak and hole 0.46 and −0.36 e.A^−3^.

#### 3.2.6. Crystallographic Data for 4-(3-carboxypropylamino)-6-chloro-5-nitrobenzo[*c*][1,2,5]oxadiazole 1-oxide (**4l**)

C_10_H_9_ClN_4_O_6_, M 316.66, monoclinic, *P2_1_/n*, *a* 11.800(6), *b* 7.300(4), *c* 15.081(7) Å, *β* 110.697(5)°, *V* 1215.2(11)Å^3^, *Z* 4, *D_calcd_* 1.731 g·cm^−3^, *μ*(Mo-*Kα*) 0.353 mm^−1^, F(000) 648, (θ 1.9-28.7°, completeness 99.0%), T 150(2) K, red-orange prism, (0.1 × 0.01 × 0.15) mm^3^, transmission 0.6572–0.7458, 10997 measured reflections in index range: −15 ≤ h ≤ 15, −9 ≤ k ≤ 9, −20 ≤ l ≤ 20, 3118 independent (*R_int_* 0.053), 193 parameters, *R_1_* = 0.0423 (for 2070 observed *I* > 2σ(*I*)), *wR_2_* = 0.1453 (all data), GOOF 0.86, largest diff. peak and hole 0.32 and −0.25 e.A^−3^.

Crystallographic data for the structures of compounds **1a**, **1b**, **4a**, **4e**, **4g**, and **4l** have been deposited at the Cambridge Crystallographic Data Centre (CCDC) where the supplementary publication no. is CCDC 2112027-2112032, accordingly. A copy of the data can be obtained, free of charge, on application to the CCDC, 12 Union Road, Cambridge CB21EZ, UK (fax: +44 122 3336033 or e-mail: deposit@ccdc.cam.ac.uk; internet: www.ccdc.cam.ac.uk (accessed on 1 March 2021)).

### 3.3. Computational Details

All reported DFT computations were performed with the Gaussian 16 series of programs [75] using the M06-2X functional [76] and the 6-311++G** basis set [77]. The geometries of the various critical points on the potential energy surface were fully optimized with the gradient method available in Gaussian 16, and harmonic vibrational frequencies were computed to evaluate the nature of all critical points. The solvent effect was considered during optimization and frequency calculations using the Polarizable Continuum Model (PCM) employing the integral equation formalism variant (IEFPCM) [78] and using methanol (ε = 32.613) [78] was used as a solvent for the calculation of the energetic.

## 4. Conclusions

Starting from some kinetic results collected studying the reactivity of 4,6-dichloro-5-ntrobenzofuroxan (**1**) with several nitrogen nucleophiles [7], we enlarged our interest to the study of the X-ray structure of **1** and of some of its amino derivatives (**4a**, **4e**, **4g**, and **4l**), gaining interesting results concerning their geometry.

As expected, different torsional angles were observed for the nitro group in **1** and in the amino derivatives (**4a**, **4e**, **4g**, and **4l**) strictly depending on the steric hindrance of the substituent at C_4_. Moreover, the results concerning the structure of **1** (that exists in two different polymorphs (**1a** and **1b**) as a function of the solvent used for the crystallization) were also confirmed: (a) the role of the two chlorine atoms in determining the out-of-plane rotation of the nitro group (so causing a sort of secondary steric effect) [61] and (b) the non-aromatic character of the “benzene” ring in **1** caused by the condensed furoxan ring.

Finally, the calculations of the energy profile concerning the nucleophilic substitution of **1** with pyrrolidine (giving **4g**) furnished a calculated profile of reaction strictly recalling the experimental kinetic results collected by some of us [7], giving a further confirmation of the “non-aromatic” character of the nucleophilic substitution occurring in **1**. 

On the whole, we can say that X-rays and DFT calculations provided a nice confirmation to the hypotheses that we had previously advanced on the basis of kinetic results [7].

## Figures and Tables

**Table 1:** C_4_–C_5_ and C_5_–N_NO2_ bond lengths (Angstrom) during the reactive process.

	d(C4–C5) [Å]	d(C5–NNO2) [Å]
RC	1.36	1.48
TS1	1.39	1.46
IC	1.49	1.38
TS2	1.50	1.39
PC	1.41	1.45

## Data Availability

The data presented in this study are contained within the article or in Supplementary Materials, or are available on request from the corresponding author Elena Chugunova.

## Supplementary Materials

The following are available online at https://www.mdpi.com/article/10.3390/ijms222413460/s1.
