# Supplementary material for: 4,6-Dichloro-5-Nitrobenzofuroxan: Different Polymorphisms and DFT Investigation of Its Reactivity with Nucleophiles
Source: Int J Mol Sci. 2021 Dec 15;22(24):13460. doi: 10.3390/ijms222413460 (PMC8709177; doi:10.3390/ijms222413460)
Supplement: Supplementary file 1 [file ijms-22-13460-s001.zip › ijms-1456441-supplementary.pdf]

Supporting Information

4,6-Dichloro-5-nitrobenzofuroxan: different polymorphisms and DFT investigation of its reactivity with nucleophiles

Elena Chugunova,[1,2*] Nurgali Akylbekov,[3] Alexey Dobrynin,[1,4] Alexander Burilov,[1,2] Carla Boga,[5] Gabriele Micheletti,[5,*] Vincenzo Frenna,[6] Edoardo Jun Mattioli,[7] Matteo Calvaresi,[7] Domenico Spinelli[7,*]

[1] Arbuzov Institute of Organic and Physical Chemistry, FRC Kazan Scientific Center, Russian Academy of Sciences, Akad. Arbuzov st. 8, Kazan, Tatarstan 420088, Russia. chugunova.e.a@gmail.com (EC); burilov_2004@mail.ru (AB); aldo@iopc.ru (AD)

[2] Laboratory of Plant Infectious Diseases, FRC Kazan Scientific Center of Russian Academy of Sciences, Lobachevskogo st. 2/31, Kazan, Tatarstan 420111 Russia.

[3] Laboratory of Engineering Profile "Physical and Chemical Methods of Analysis", Korkyt Ata Kyzylor-da University, Aitekebie str. 29A, Kyzylorda 120014, Kazakhstan. nurgali_089@mail.ru (NA)

[4] Kazan National Research Technical University, 10 Karl Marx Str., Kazan, 420111, Russia.

[5] Department of Industrial Chemistry 'Toso Montanari' ALMA MATER STUDIORUM – Università di Bologna, Viale del Risorgimento 4, 40136 Bologna, Italy. carla.boga@unibo.it (CB); gabriele.micheletti3@unibo.it (GM).

[6] Department STEBICEF, University of Palermo, Ed.17, Viale delle Scienze 90128 Palermo. vincenzo.frenna@unipa.it (VF)

[7] Department of Chemistry 'G. Ciamician' ALMA MATER STUDIORUM – Università di Bologna, Via Selmi 2, 40126 Bo-logna, Italy. edoardojun.mattioli2@unibo.it (EJM) matteo.calvaresi3@unibo.it (MC); domenico.spinelli@unibo.it (DS).

[*] Correspondence: chugunova.e.a@gmail.com (EC); gabriele.micheletti3@unibo.it (GM); domenico.spinelli@unibo.it (DS)

Content **Page**

Figure S1. Rotational barrier of the nitrogroup in **(2), (11), (12)** S3

Figure S2. Schematic two-dimensional and three-dimensional representation of

the reactant complex RC, transition state TS1 and, intermediate structure IC. S4

Figure S3. Schematic two-dimensional (top) and three-dimensional (bottom) representation of the intermediate complex IC, transition state TS2 and, product complex PC. S4

Figure S4. Thermal ellipsoid plot for compound **1a**. S5

Figure S5. Thermal ellipsoid plot for compound **1b**. S5

Figure S6. Thermal ellipsoid plot for compound **4a**. S6

Figure S7. Thermal ellipsoid plot for compound **4e**. S6

Figure S8. Thermal ellipsoid plot for compound **4g**. S7

Figure S9. Thermal ellipsoid plot for compound **4l**. S7

Cartesian coordinates, the number of imaginary frequencies, and computed total energies and free energies of the optimized structures discussed in the manuscript S8-S19

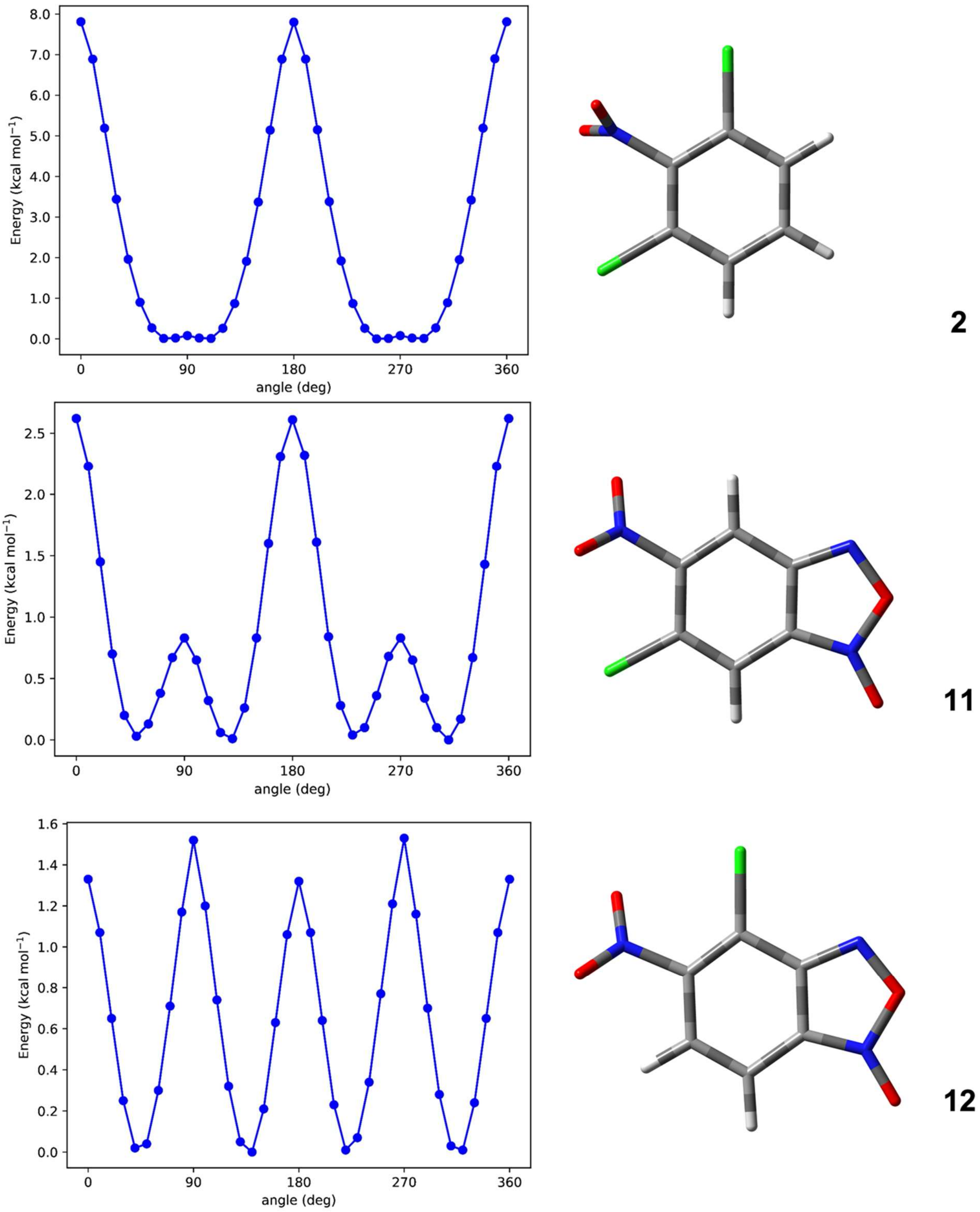

Figure S1. Rotational barrier of the nitro group in **(2), (11), (12)**

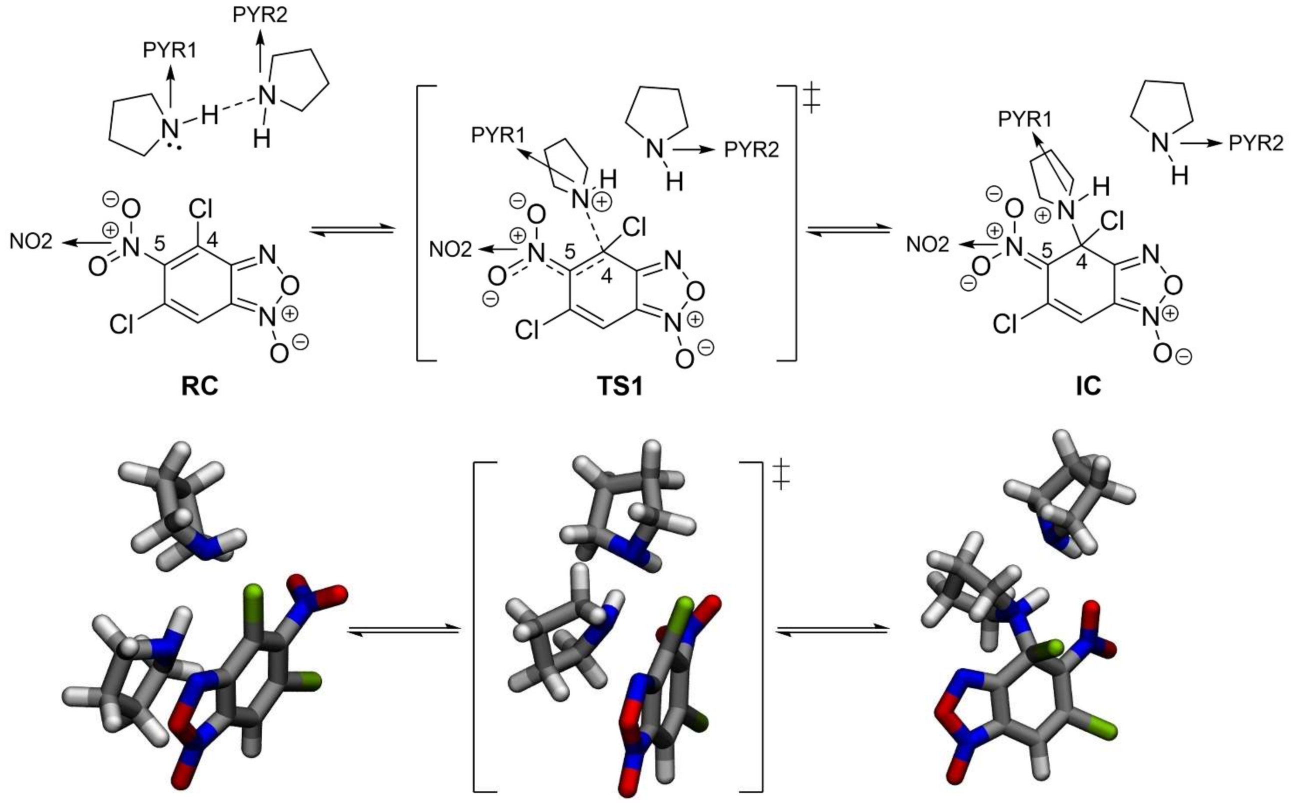

Figure S2. Schematic two-dimensional (top) and three-dimensional (bottom) representation of the reactant complex RC, transition state TS1 and, intermediate structure IC.

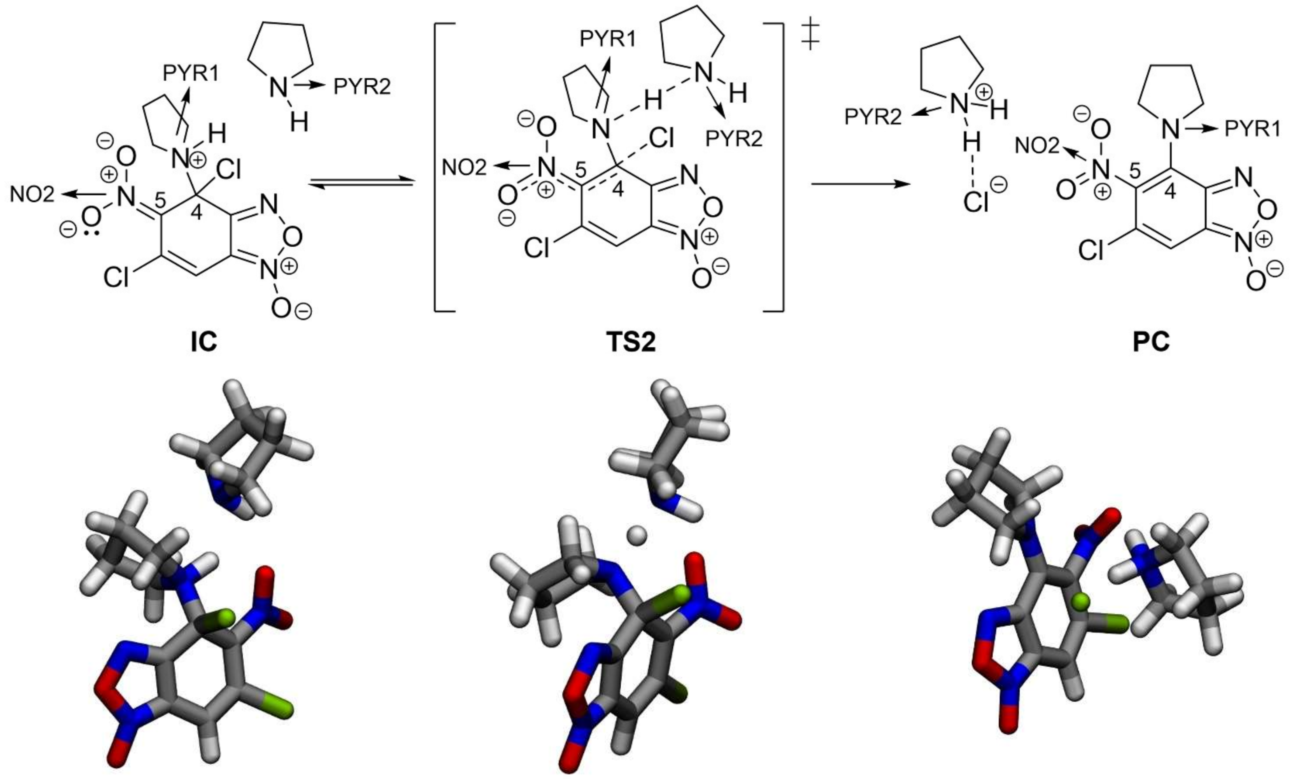

Figure S3. Schematic two-dimensional (top) and three-dimensional (bottom) representation of the intermediate complex IC, transition state TS2 and, product complex PC.

Datablock chug3 - ellipsoid plot

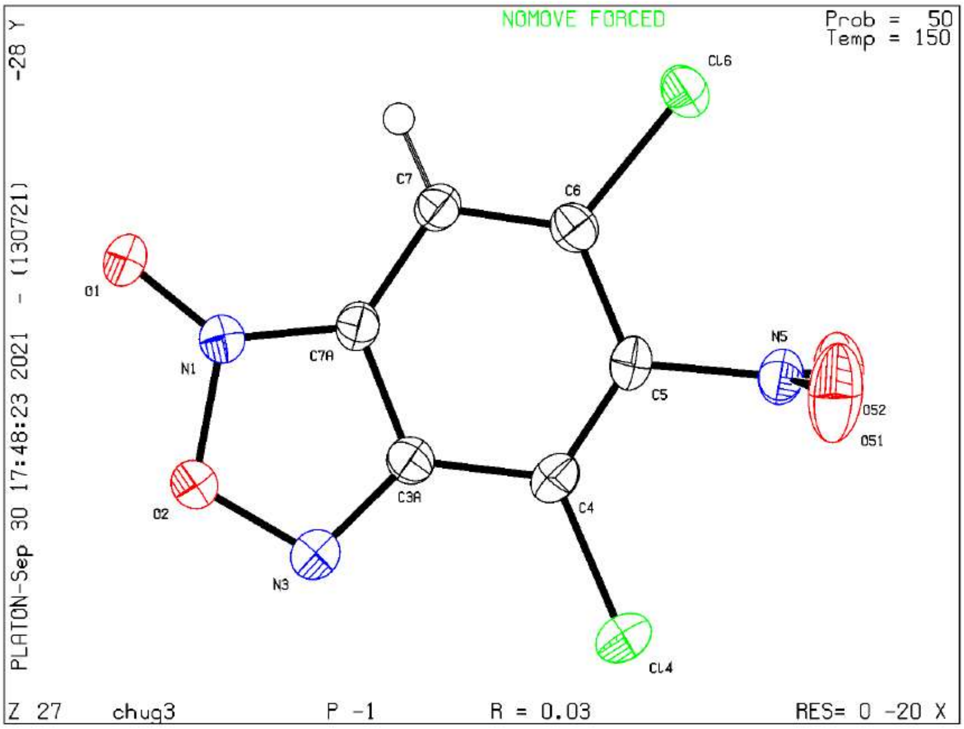

Figure S4. Thermal ellipsoid plot for compound **1a**.

Datablock chug8 - ellipsoid plot

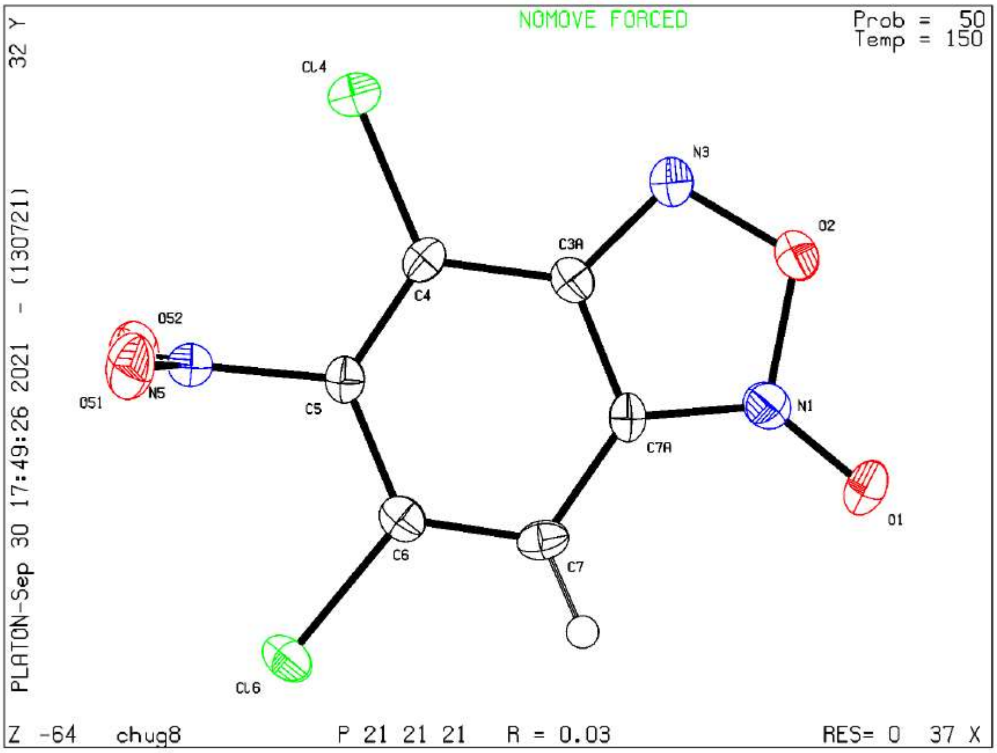

Figure S5. Thermal ellipsoid plot for compound **1b**.

Datablock nur25_fin - ellipsoid plot

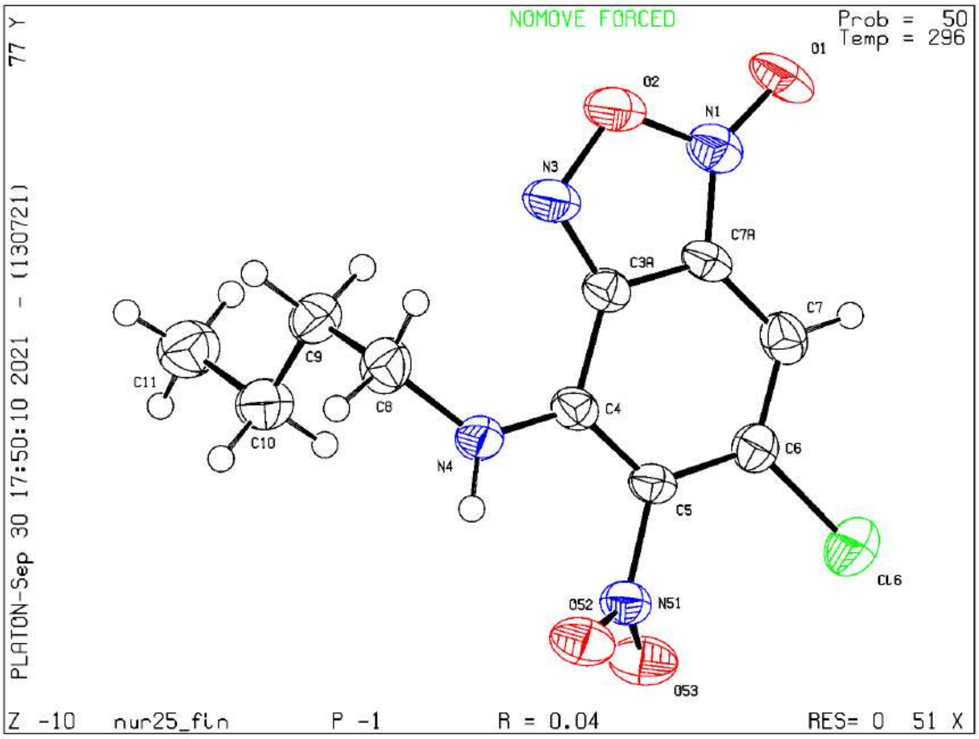

Figure S6. Thermal ellipsoid plot for compound **4a**.

Datablock nur27_new - ellipsoid plot

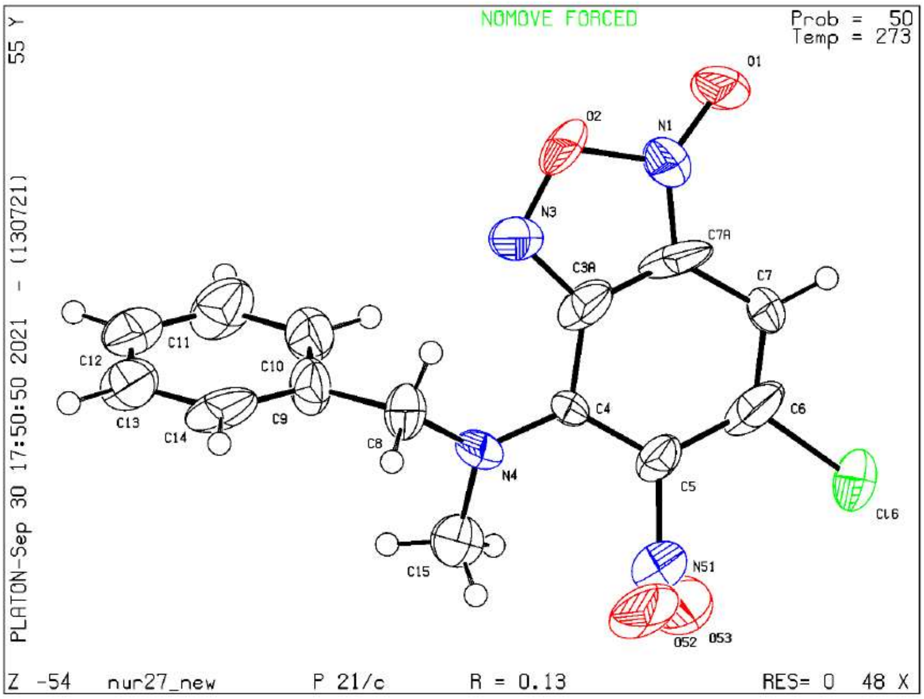

Figure S7. Thermal ellipsoid plot for compound **4e**.

Datablock nurgali1 - ellipsoid plot

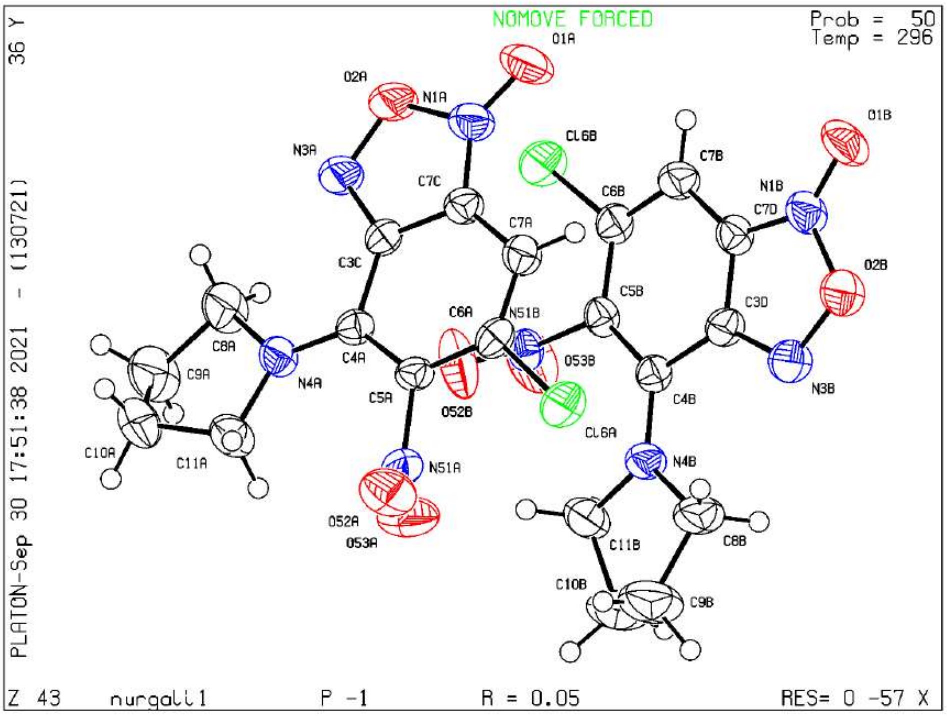

Figure S8. Thermal ellipsoid plot for compound **4g**.

Datablock chug4_fin - ellipsoid plot

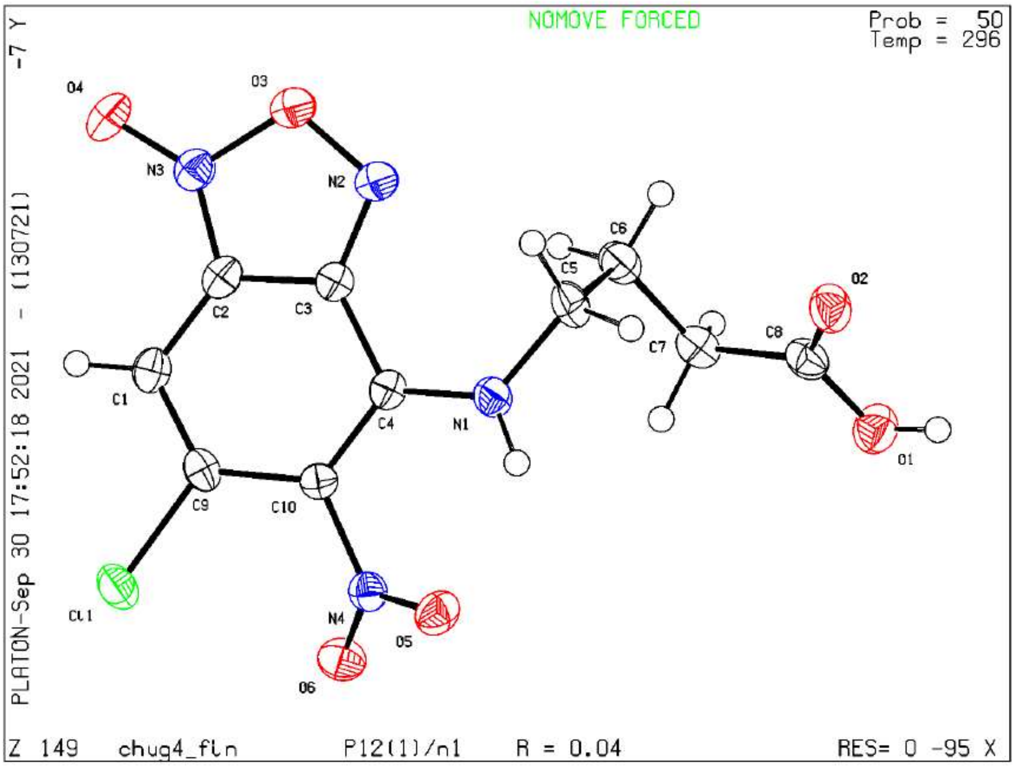

Figure S9. Thermal ellipsoid plot for compound **4l**.

Cartesian coordinates, the number of imaginary frequencies, and computed total energies and free energies of the optimized structures discussed in the manuscript

Min from 1a

C	-0.062327	0.010451	-0.032941
C	0.025413	0.008716	1.319378
C	1.269094	0.012061	2.057232
C	2.462563	0.009716	1.412751
C	2.390522	-0.001286	-0.000784
C	1.182816	-0.000176	-0.741900
N	-1.230157	-0.014986	2.099262
Cl	1.199459	0.055189	3.779272
H	3.408524	0.022320	1.936196
N	3.364516	-0.010920	-0.911575
N	1.407157	-0.009793	-2.030331
Cl	-1.536894	-0.003775	-0.894866
O	2.755857	-0.016013	-2.159104
O	4.578879	-0.015472	-0.854830
O	-1.858780	1.013186	2.158455
O	-1.523393	-1.065657	2.615204

E(RM062X) = -1614.43337818 A.U.
Sum of electronic and thermal Free Energies= -1614.392442 A.U.

Min from 1b

C	-0.061703	-0.042638	-0.032554
C	0.025544	-0.011671	1.319448
C	1.268916	0.017921	2.057140
C	2.462453	0.024966	1.412892
C	2.390526	0.007142	-0.000549
C	1.183296	-0.027146	-0.741460
N	-1.230397	0.009594	2.099018
Cl	1.199077	0.007031	3.779802
H	3.408274	0.036491	1.936631
N	3.364431	0.014274	-0.911697
N	1.407749	-0.038574	-2.029913
Cl	-1.536109	-0.069492	-0.894480
O	2.756393	-0.014130	-2.158874
O	4.578485	0.038865	-0.855132
O	-1.539147	1.066170	2.593273
O	-1.843471	-1.026367	2.179613

E(RM062X) = -1614.43337173 A.U.
Sum of electronic and thermal Free Energies= -1614.392430 A.U.

1TS180

C	-0.067874	-0.000071	-0.022056
C	-0.019162	-0.000097	1.353238
C	1.266379	-0.000062	2.070206
C	2.451854	-0.000017	1.406457
C	2.396457	-0.000000	0.000157
C	1.187524	-0.000025	-0.720530

N	-1.298876	-0.000148	2.118082
Cl	1.421193	-0.000069	3.796163
H	3.395510	0.000007	1.933215
N	3.365938	0.000042	-0.914293
N	1.399711	0.000002	-2.014084
Cl	-1.423496	-0.000082	-1.062908
O	2.742574	0.000045	-2.156949
O	4.580997	0.000078	-0.869054
O	-1.245847	0.000192	3.324023
O	-2.331506	0.000205	1.489839

E(RM062X) = -1614.41879034 A.U.
Sum of electronic and thermal Free Energies = -1614.377560 A.U.
1 imaginary frequency -114.820 cm-1

1TS90

C	-0.049611	0.223144	-0.017674
C	0.027495	0.004554	1.316328
C	1.255620	-0.237920	2.037216
C	2.448807	-0.263854	1.392699
C	2.388682	-0.038525	-0.003659
C	1.194627	0.204393	-0.727638
N	-1.228283	0.007641	2.097431
Cl	1.157369	-0.490693	3.739593
H	3.384967	-0.442421	1.903386
N	3.365832	0.000453	-0.910671
N	1.430281	0.381143	-2.001849
Cl	-1.515303	0.506396	-0.848110
O	2.773133	0.258806	-2.138208
O	4.572630	-0.136524	-0.862833
O	-1.795281	-1.050541	2.216310
O	-1.579890	1.066747	2.555589

E(RM062X) = -1614.43307171 A.U.
Sum of electronic and thermal Free Energies = -1614.391032 A.U.
1 imaginary frequency -114.820 cm-1

2

C	-0.026557	-0.030262	0.010781
C	-0.035891	-0.025199	1.398363
C	1.170269	0.000324	2.088091
C	2.384788	0.025610	1.413174
C	2.392567	0.030121	0.025592
C	1.187206	-0.000262	-0.663470
H	-0.981092	-0.040074	1.924245
H	1.163683	0.000555	3.170442
H	3.323392	0.040798	1.950713
Cl	3.890759	0.044759	-0.840716
N	1.196279	-0.000488	-2.138005
Cl	-1.513785	-0.044887	-0.874187
O	0.935647	-1.043404	-2.690525

O	1.463692	1.042267	-2.687586

E(RM062X) = -1355.87740648 A.U.
Sum of electronic and thermal Free Energies = -1355.829200 A.U.

2TS180

C	-0.030785	-0.000003	0.000654
C	-0.023635	0.000000	1.389902
C	1.170506	0.000002	2.087672
C	2.372468	0.000000	1.402592
C	2.394409	-0.000003	0.013720
C	1.185575	-0.000005	-0.716734
H	-0.970795	0.000001	1.911631
H	1.164878	0.000004	3.170029
H	3.313450	0.000001	1.935177
Cl	3.989559	-0.000004	-0.667774
N	1.193554	-0.000008	-2.197539
Cl	-1.620126	-0.000004	-0.696322
O	0.131534	0.000009	-2.778812
O	2.261836	0.000009	-2.767384

E(RM062X) = -1355.86495883 A.U.
Sum of electronic and thermal Free Energies= -1355.815601 A.U.
1 imaginary frequency -109.51 cm-1

2TS90

C	0.001575	-0.265833	0.013770
C	-0.006456	-0.268054	1.401379
C	1.172346	-0.003404	2.088783
C	2.357134	0.263725	1.412614
C	2.361433	0.266414	0.025031
C	1.184473	0.001527	-0.659859
H	-0.927872	-0.475646	1.928764
H	1.167779	-0.005268	3.171135
H	3.273686	0.469444	1.949116
Cl	3.814073	0.596281	-0.856048
N	1.190887	0.003801	-2.135483
Cl	-1.443027	-0.592220	-0.881700
O	0.956385	1.054225	-2.684714
O	1.429953	-1.044971	-2.685974

E(RM062X) = -1355.87727020 A.U.
Sum of electronic and thermal Free Energies= -1355.828010 A.U.
1 imaginary frequency -48.27 cm-1

11

C	-0.091249	0.053345	-0.030284
C	0.004612	0.011731	1.316990
C	1.251840	-0.006045	2.057812
C	2.437412	-0.016163	1.397538

C	2.361392	-0.016119	-0.016589
C	1.145810	0.030424	-0.744686
N	-1.276650	-0.059559	2.051270
Cl	1.240378	0.085694	3.780701
H	3.387648	-0.002714	1.913627
N	3.329865	-0.030539	-0.929799
N	1.366712	0.037382	-2.036572
H	-1.046151	0.080857	-0.538073
O	2.714023	0.000866	-2.174125
O	4.546771	-0.063700	-0.882554
O	-2.126065	0.751359	1.761698
O	-1.395609	-0.947339	2.862508

E(RM062X) = -1154.84419418 A.U.
Sum of electronic and thermal Free Energies= -1154.791313 A.U.

11TS180

C	-0.076268	-0.061501	-0.007638
C	-0.000069	-0.004927	1.345342
C	1.265193	0.059865	2.072679
C	2.441389	0.066469	1.389223
C	2.368765	0.008102	-0.020242
C	1.149673	-0.055144	-0.732897
N	-1.323561	-0.015265	2.036781
Cl	1.386289	0.131497	3.795808
H	3.393079	0.114279	1.900212
N	3.329993	0.001262	-0.941784
N	1.355838	-0.099717	-2.028065
H	-1.029231	-0.108756	-0.514385
O	2.699493	-0.065879	-2.179132
O	4.547115	0.039076	-0.908902
O	-1.347498	0.034440	3.242151
O	-2.309094	-0.073555	1.335425

E(RM062X) = -1154.84003221 A.U.
Sum of electronic and thermal Free Energies= -1154.786498 A.U.
1 imaginary frequency -80.390 cm-1

11TS90

C	-0.087247	0.247427	-0.039361
C	-0.002788	0.012273	1.285745
C	1.219185	-0.248090	2.014766
C	2.412076	-0.274567	1.370570
C	2.355629	-0.032753	-0.024326
C	1.158931	0.227656	-0.741918
N	-1.257017	0.011921	2.071660
Cl	1.119436	-0.520076	3.715179
H	3.347180	-0.465745	1.879106
N	3.334118	0.009441	-0.926414
N	1.403082	0.417494	-2.015155
H	-1.026028	0.437631	-0.542507

O	2.746859	0.286492	-2.150976
O	4.542714	-0.137599	-0.878779
O	-1.831930	-1.044311	2.181085
O	-1.603127	1.065606	2.549234

E(RM062X) = -1154.84287106 A.U.
Sum of electronic and thermal Free Energies= -1154.789420 A.U.
1 imaginary frequency -66.610 cm-1

12

C	-0.074436	0.004536	-0.020298
C	0.007255	0.010984	1.338179
C	1.238540	0.003569	2.084228
C	2.439770	0.013942	1.457143
C	2.390687	0.011148	0.040481
C	1.186244	-0.001852	-0.706841
N	-1.220132	-0.007085	2.156803
H	1.169527	-0.004390	3.163332
H	3.380402	0.021246	1.989554
N	3.369215	0.004424	-0.864325
N	1.420214	-0.016761	-1.995032
Cl	-1.502237	-0.053079	-0.956681
O	2.769260	-0.011672	-2.115905
O	4.584413	0.008997	-0.801684
O	-1.214878	-0.743383	3.118283
O	-2.132426	0.715503	1.832027

E(RM062X) = -1154.84491856 A.U.
Sum of electronic and thermal Free Energies= -1154.791704

12TS180

C	-0.078213	-0.073941	-0.020389
C	-0.006220	-0.004758	1.344161
C	1.237075	0.074286	2.073713
C	2.439508	0.086149	1.451599
C	2.396029	0.015587	0.037953
C	1.190576	-0.062463	-0.699403
N	-1.223714	-0.007572	2.190220
H	1.173004	0.124950	3.150160
H	3.376043	0.145625	1.987707
N	3.371388	0.008239	-0.869110
N	1.420532	-0.115714	-1.988184
Cl	-1.455741	-0.169870	-1.024168
O	2.766017	-0.073171	-2.116837
O	4.586425	0.055367	-0.813358
O	-1.059830	0.056752	3.389472
O	-2.303956	-0.073283	1.651627

E(RM062X) = -1154.84278504 A.U.
Sum of electronic and thermal Free Energies= -1154.788451
1 imaginary frequency -66.830 cm-1

12TS90

C	-0.049676	0.244629	0.018188
C	0.030178	0.003157	1.348753
C	1.238048	-0.268101	2.076637
C	2.431498	-0.302220	1.431582
C	2.386239	-0.055959	0.035959
C	1.196092	0.214608	-0.688102
N	-1.221480	0.019212	2.136051
H	1.170434	-0.442919	3.142533
H	3.365627	-0.502275	1.937640
N	3.364869	-0.020731	-0.869715
N	1.438693	0.404438	-1.960110
Cl	-1.511033	0.566929	-0.811330
O	2.781892	0.262482	-2.093995
O	4.570042	-0.177814	-0.821353
O	-1.812730	-1.028314	2.243423
O	-1.547619	1.075677	2.621748

E(RM062X) = -1154.84246969 A.U.
Sum of electronic and thermal Free Energies= -1154.788663
1 imaginary frequency -76.800 cm-1

Isolated reactants
Pyr

N	0.121592	-0.089953	-0.019678
C	0.013544	-0.092964	1.445239
C	1.442545	0.121861	1.983009
C	2.151301	0.913120	0.855807
C	1.108418	0.966429	-0.288426
H	-0.628576	0.743840	1.737763
H	-0.450789	-1.014696	1.796764
H	1.929467	-0.843351	2.136805
H	1.447911	0.651556	2.936553
H	3.056663	0.398252	0.530552
H	2.440036	1.915331	1.175494
H	1.545479	0.838467	-1.278881
H	0.585827	1.927271	-0.272913
H	0.521557	-0.979757	-0.308011

E(RM062X) = -212.541222850 A.U.
Sum of electronic and thermal Free Energies= -212.439484 A.U.

1

C	-0.061703	-0.042638	-0.032554
C	0.025544	-0.011671	1.319448
C	1.268916	0.017921	2.057140
C	2.462453	0.024966	1.412892
C	2.390526	0.007142	-0.000549
C	1.183296	-0.027146	-0.741460

N	-1.230397	0.009594	2.099018
Cl	1.199077	0.007031	3.779802
H	3.408274	0.036491	1.936631
N	3.364431	0.014274	-0.911697
N	1.407749	-0.038574	-2.029913
Cl	-1.536109	-0.069492	-0.894480
O	2.756393	-0.014130	-2.158874
O	4.578485	0.038865	-0.855132
O	-1.539147	1.066170	2.593273
O	-1.843471	-1.026367	2.179613

E(RM062X) = -1614.43337173 A.U.
Sum of electronic and thermal Free Energies= -1614.392430 A.U.

RC

C	-1.346981	5.234340	2.682450
N	-1.014686	3.953592	3.327291
C	-0.462358	4.346609	4.630609
C	-1.328616	5.520558	5.115329
C	-1.765537	6.219355	3.805799
O	-1.135831	1.014568	4.126992
N	-1.335157	0.504520	3.050673
O	-2.330261	-0.097182	2.722650
C	-0.262442	0.633194	2.043572
C	-0.503632	1.379973	0.938888
C	0.589938	1.521648	0.022893
C	1.813028	0.864694	0.304301
C	2.041096	0.068955	1.451917
C	0.998538	-0.027055	2.312243
N	0.660711	2.219167	-1.078616
O	1.927387	2.045079	-1.534574
N	2.636431	1.207622	-0.677332
N	1.711471	3.260781	2.074993
C	2.822997	3.027002	2.991981
C	4.032868	3.034794	2.047151
C	3.626356	4.028881	0.928275
C	2.153425	4.373274	1.235722
Cl	-1.973114	2.187249	0.612599
Cl	1.143721	-0.993361	3.735826
O	3.808523	0.973859	-0.930111
H	2.984991	-0.429968	1.624214
H	1.533038	4.454990	0.338843
H	2.103485	5.334738	1.770284
H	4.247195	4.925177	0.931798
H	3.729765	3.570092	-0.057173
H	4.951248	3.326039	2.557265
H	4.189993	2.036877	1.629131
H	2.926533	3.834394	3.735142
H	2.699954	2.083526	3.529722

H	-1.873231	3.432903	3.489268
H	-0.443942	3.494940	5.311069
H	0.570423	4.680881	4.471831
H	-0.790043	6.186455	5.790697
H	-2.198961	5.134059	5.650421
H	-1.273443	7.184654	3.679090
H	-2.841874	6.395396	3.794797
H	-0.446525	5.600056	2.178326
H	-2.116102	5.087810	1.924135
H	0.819066	3.439463	2.537671

SCF Done: E(RM062X) = -2039.54082941 A.U.
Sum of electronic and thermal Free Energies= -2039.256393 A.U.

TS1

C	-3.461707	4.429269	2.282428
N	-2.335743	3.955434	3.101475
C	-1.901340	5.145031	3.861001
C	-3.141625	6.063631	4.031981
C	-4.244196	5.385507	3.190576
O	-1.404224	1.137567	3.867473
N	-1.753371	0.927456	2.722058
O	-2.905994	0.953532	2.344702
C	-0.696992	0.673387	1.744823
C	-0.606485	1.533949	0.658986
C	0.542212	1.298189	-0.194776
C	1.535719	0.360494	0.184759
C	1.436726	-0.486022	1.319440
C	0.326426	-0.295452	2.075121
N	0.839732	1.881231	-1.323511
O	2.028363	1.358899	-1.705966
N	2.453583	0.410926	-0.771530
N	0.091239	3.423324	1.533837
C	1.295209	3.203607	2.348228
C	2.422116	3.774570	1.489891
C	1.760515	5.017443	0.886641
C	0.331114	4.538828	0.593656
Cl	-1.970822	2.297454	-0.097614
Cl	0.063904	-1.363951	3.413985
O	3.511494	-0.170210	-0.974924
H	2.168635	-1.251102	1.530433
H	0.246577	4.169166	-0.433552
H	-0.418747	5.321185	0.728249
H	1.742169	5.819070	1.629444
H	2.264125	5.387297	-0.006082
H	3.318209	3.996362	2.068332
H	2.693454	3.067277	0.699461
H	1.220598	3.747307	3.297557
H	1.406957	2.141924	2.585199

H	-2.685735	3.257865	3.754166
H	-1.454986	4.839052	4.807221
H	-1.127481	5.658633	3.280493
H	-2.926112	7.067717	3.663779
H	-3.434152	6.152724	5.078471
H	-4.849896	6.099960	2.631950
H	-4.911937	4.805916	3.832813
H	-3.054417	4.967579	1.418958
H	-4.042517	3.583906	1.913046
H	-0.757080	3.562177	2.104882

E(RM062X) = -2039.53142692 A.U.
Sum of electronic and thermal Free Energies= -2039.243094 A.U.
1 imaginary frequency -163.1648 cm -1

IC

C	-2.903998	2.135407	0.936957
N	-1.591046	2.444177	1.536376
C	-1.601315	3.914741	1.643891
C	-3.046176	4.316274	2.015865
C	-3.905293	3.094442	1.604218
O	-0.397017	-2.154830	2.609374
N	-0.511342	-1.280003	1.748787
O	-1.063504	-0.191825	2.012584
C	0.001090	-1.483256	0.481313
C	0.192054	-0.328632	-0.443265
C	1.317840	-0.687672	-1.377804
C	1.587152	-2.042422	-1.695432
C	0.894072	-3.144483	-1.118476
C	0.148472	-2.829732	-0.033174
N	2.134465	0.109167	-1.996433
O	2.946970	-0.671458	-2.742096
N	2.592172	-2.017931	-2.549134
N	0.427354	0.999832	0.197999
C	1.647418	1.024985	1.113077
C	2.512975	2.166235	0.592056
C	1.489097	3.131917	-0.004333
C	0.510597	2.213229	-0.723256
Cl	-1.329984	-0.074010	-1.517921
Cl	-0.817227	-4.124851	0.620881
O	3.229967	-2.872094	-3.154383
H	0.935874	-4.134968	-1.544813
H	0.877108	1.909687	-1.696893
H	-0.496192	2.613145	-0.813455
H	0.977684	3.678897	0.790379
H	1.927635	3.849886	-0.695811
H	3.099922	2.608449	1.395407
H	3.191268	1.803654	-0.181158
H	1.263540	1.219498	2.114545
H	2.129720	0.049757	1.098528

H	-1.584503	2.052815	2.477042
H	-0.856682	4.249514	2.366325
H	-1.336142	4.326799	0.665111
H	-3.347378	5.227546	1.498470
H	-3.127542	4.503182	3.087422
H	-4.719720	3.362343	0.930733
H	-4.343918	2.620921	2.484415
H	-2.838055	2.339964	-0.136773
H	-3.137119	1.079254	1.068150
H	-0.406070	1.248276	0.802659

E(RM062X) = -2039.56096264 A.U.
Sum of electronic and thermal Free Energies= -2039.268235 A.U.

TS2

C	2.071554	2.423012	2.746763
N	0.861907	2.625406	1.892359
C	1.348912	3.594380	0.854914
C	2.826690	3.240768	0.594863
C	3.220210	2.290089	1.749402
C	0.249870	1.334981	1.368451
C	0.295995	0.190372	2.329804
C	1.101505	-0.984319	2.109930
C	1.742992	-1.277545	0.946972
C	1.521945	-0.342995	-0.099306
C	0.827204	0.882367	0.051076
N	0.733524	1.510338	-1.087699
O	1.386154	0.756486	-2.000351
N	1.870974	-0.407913	-1.374501
N	-0.447013	0.351415	3.496885
O	-0.734232	1.516009	3.842573
Cl	1.470125	-2.058326	3.436135
O	2.487436	-1.221685	-2.059359
O	-0.825370	-0.620341	4.153742
Cl	-1.502068	1.843294	0.967635
H	2.401921	-2.124944	0.826901
H	0.703051	3.570763	-0.020995
H	1.259637	4.579235	1.321292
H	3.429795	4.150230	0.603350
H	2.957517	2.765646	-0.379601
H	4.175836	2.551449	2.206141
H	3.293453	1.253539	1.403125
H	2.166136	3.335292	3.343613
H	1.929821	1.577541	3.416495
H	-1.619384	3.482832	3.350228
N	-0.845636	4.130476	3.182924
C	-0.380301	4.634720	4.494488
C	-1.411485	5.701607	4.915880
C	-2.114358	6.114895	3.596395

C	-1.369428	5.333038	2.501281
H	-0.278679	3.800951	5.190880
H	0.604624	5.088101	4.343639
H	-0.922501	6.545800	5.406083
H	-2.131515	5.281202	5.621723
H	-2.073682	7.191123	3.417501
H	-3.166914	5.820680	3.614624
H	-0.514427	5.907073	2.130577
H	-1.990099	5.043600	1.651310
H	0.058200	3.240516	2.507899

E(RM062X) = -2039.56079866 A.U.
Sum of electronic and thermal Free Energies= -2039.268371 A.U.
1 imaginary frequency -413.7329 cm-1

PC

C	0.291357	3.745805	0.732107
N	1.080286	2.598459	0.246848
C	1.796626	2.951144	-1.001482
C	1.670248	4.467566	-1.065363
C	0.291711	4.705964	-0.452035
C	0.905255	1.348310	0.658771
C	0.237669	0.948686	1.835057
C	-0.181433	-0.410094	2.092102
C	0.068635	-1.434798	1.240667
C	0.841357	-1.085271	0.107449
C	1.310766	0.222372	-0.185356
N	2.055250	0.213631	-1.260453
O	2.089039	-1.064421	-1.689665
N	1.332647	-1.867930	-0.839295
N	-0.001137	1.937512	2.865597
O	-1.124362	2.035896	3.344455
Cl	-0.938624	-0.816331	3.604016
O	1.262719	-3.069618	-1.080656
O	0.934907	2.627302	3.218261
Cl	-1.632049	1.247879	-1.254938
H	-0.219683	-2.454970	1.444687
H	2.823058	2.591853	-0.965409
H	1.278016	2.478115	-1.840117
H	1.758710	4.831220	-2.087775
H	2.445082	4.939653	-0.456765
H	-0.490052	4.420073	-1.159294
H	0.127415	5.734047	-0.133212
H	-0.709771	3.401436	1.002497
H	0.770017	4.194662	1.606223
H	-2.433408	1.013261	0.592231
N	-3.116471	0.885941	1.380159
C	-3.664084	-0.521399	1.298131
C	-5.144483	-0.348314	0.943273
C	-5.201978	1.030257	0.278858

C	-4.245589	1.847544	1.134392
H	-3.106275	-1.027934	0.510652
H	-3.495220	-1.026967	2.245329
H	-5.751286	-0.352144	1.850623
H	-5.491318	-1.150141	0.293880
H	-6.202368	1.459588	0.268167
H	-4.825720	0.979808	-0.745755
H	-4.685322	2.106938	2.096934
H	-3.842054	2.736841	0.653667
H	-2.632326	1.085391	2.260612

E(RM062X) = -2039.62262111 A.U.
Sum of electronic and thermal Free Energies= -2039.329959 A.U.
